# Postprandial Glycemic and Satiety Responses to Cooked Quinoa: A Randomized Crossover Preliminary Study

**DOI:** 10.3390/foods15152610

**Published:** 2026-07-26

**Authors:** Tasleem A. Zafar

**Affiliations:** Department of Food Science and Nutrition, College of Life Sciences, Kuwait University, Shadadiya 12037, Kuwait; tasleem.zafar@ku.edu.kw

**Keywords:** dietary carbohydrates, functional foods, postprandial glycemia, quinoa, satiety

## Abstract

Quinoa (*Chenopodium quinoa* Willd.) is a nutrient-dense pseudocereal with a favorable nutritional profile that may improve postprandial glycemic control and satiety. This randomized crossover pilot study compared the acute effects of cooked quinoa and white bread on postprandial blood glucose and subjective appetite responses in healthy young adults. Participants consumed four test meals on separate occasions: cooked quinoa or white bread providing either 25 g or 35 g available carbohydrate. Capillary blood glucose and appetite ratings were assessed over 120 min. Quinoa produced significantly lower postprandial glucose responses and incremental area under the curve (iAUC) than white bread at both carbohydrate doses (*p* < 0.05). Appetite responses were also improved following quinoa consumption compared to the control. Blood glucose iAUC was positively correlated with appetite net area under the curve (r = 0.57; *p* < 0.001). Palatability and gastrointestinal tolerance were comparable between treatments. These findings indicate that cooked quinoa elicits more favorable postprandial glycemic and satiety responses than white bread and may serve as a functional carbohydrate alternative to support metabolic health.

## 1. Introduction

Dietary patterns characterized by high consumption of refined carbohydrates are associated with exaggerated postprandial glycemic excursions, reduced satiety, and an increased risk of obesity and type 2 diabetes mellitus (T2DM). These concerns are particularly relevant in Kuwait, where more than 75% of adults are overweight or obese and diabetes prevalence remains among the highest worldwide [1,2,3,4,5,6]. The ongoing nutrition transition, marked by increased consumption of refined grains and reduced dietary fiber intake, has further contributed to this growing public health burden [5,6]. Consequently, identifying culturally acceptable carbohydrate alternatives that improve postprandial glycemic control while enhancing satiety has become an important nutritional strategy.

Quinoa (*Chenopodium quinoa* Willd.) is a nutrient-dense pseudocereal traditionally cultivated in South America and increasingly recognized as a functional food. Compared with commonly consumed cereals such as wheat, rice, and maize, quinoa contains higher-quality protein with a balanced essential amino acid profile, greater dietary fiber, unsaturated fatty acids, vitamins, minerals, and numerous bioactive compounds including phenolics, phytosterols, and saponins [7,8,9,10,11,12,13,14,15]. These nutritional characteristics suggest that quinoa may beneficially influence glucose metabolism and appetite regulation.

Several mechanisms have been proposed to explain these beneficial effects. The relatively high fiber content and slowly digestible starch fraction of quinoa can delay gastric emptying, carbohydrate digestion, and glucose absorption, thereby attenuating postprandial glycemic responses. In addition, its higher protein content and amino acid composition may enhance satiety signaling and prolong postprandial fullness [16,17]. Experimental studies in animal models and in vitro systems have demonstrated reductions in food intake, body weight gain, and blood glucose following quinoa supplementation [18,19,20,21,22,23,24]. However, evidence from human intervention studies remains limited and inconsistent.

Clinical trials evaluating quinoa consumption have produced mixed findings. In overweight adults, daily consumption of bread containing 20 g quinoa flour for three months improved glycemic parameters [25]. In contrast, several studies using approximately 15–25 g quinoa or quinoa flour incorporated into bread, biscuits, cereal bars, or flakes reported no significant improvements in fasting glucose or glycated hemoglobin in healthy adults [26,27,28,29]. A dose–response trial demonstrated that 50 g, but not 25 g, quinoa improved hypertriglyceridemia; however, neither dose significantly affected glycemic outcomes [30]. Overall, greater metabolic benefits have generally been observed with higher quinoa intakes and longer intervention periods, particularly when combined with lifestyle modification. For example, daily consumption of 50 g quinoa together with energy restriction improved insulin resistance in overweight adults [31]. Likewise, replacing staple cereals with approximately 100 g quinoa daily for one year improved insulin resistance and reduced glycated hemoglobin (HbA1c) [32]. Other studies reported that consuming 100 g quinoa daily as a replacement for approximately half of the staple carbohydrate foods for one year [33], or replacing carbohydrate staples entirely with quinoa or quinoa-based products for four weeks [34] or fourteen weeks [35], improved glucose tolerance and insulin resistance in individuals with prediabetes or type 2 diabetes.

The variability among these findings is likely attributable to differences in study design, quinoa dose, intervention duration, food matrix, participant characteristics, and ethnic background, all of which are known to influence dietary intervention outcomes [36]. Furthermore, the long-term safety and tolerability of consuming large quantities of quinoa have not been adequately investigated. Complete replacement of staple carbohydrate foods with quinoa may also reduce dietary adherence and limit the practicality of such interventions in free-living populations.

To our knowledge, no study has evaluated the acute effects of cooked quinoa on both postprandial glycemic responses and subjective appetite sensations in a Kuwaiti population. Simultaneous assessment of these outcomes remains scarce despite their importance for understanding the potential of quinoa as a functional carbohydrate source capable of improving both glycemic regulation and appetite control.

Therefore, this preliminary randomized crossover study evaluated the acute effects of cooked quinoa providing 25 g and 35 g available carbohydrate (equivalent to 36 g and 50 g dry quinoa seeds, respectively) on postprandial blood glucose and subjective appetite responses in healthy young female adults, using white bread as the reference food. These carbohydrate doses were selected to explore a potential dose–response relationship and to identify a minimum effective dose for future intervention studies in the Kuwaiti population. We hypothesized that cooked quinoa would produce lower postprandial glycemic responses and greater satiety than white bread, with greater effects at the higher carbohydrate dose.

## 2. Methods

### 2.1. Study Design and Participants

This acute, randomized, crossover study was conducted in the Human Nutrition Laboratories, Department of Food Science and Nutrition, Kuwait University, during February and March 2024. Healthy male and female students (aged 18–30 years; BMI 20–25 kg/m^2^) were invited to participate in the study via flyers and word of mouth. Only female students responded to participate. Out of 31 interested students, 18 were eligible by the inclusion/exclusion criteria. Inclusion criteria as given above was advertised in the flyers. Exclusion criteria applied included BMI or age deviation, elevated fasting blood glucose, use of medication affecting glucose metabolism or appetite, restrained eating behavior (score ≥ 11 on the Eating Habits Questionnaire), or habitual breakfast skipping [37].

The study protocol was approved by the Human Subjects’ Review Committee, Ethics Review Office, Kuwait University, and written informed consent was obtained from all participants. Sample size estimation was based on previous acute glycemic response studies with an α of 0.05 and power of 0.80 [38,39,40,41].

### 2.2. Test Foods

Test foods included cooked quinoa (Bob’s Red Mill, Natural Foods, Milwaukie, OR, USA) and white wheat bread (Kuwait Flour Mills & Bakeries Co., Kuwait City, Kuwait). Portions of quinoa and white bread were matched to provide either 25 g or 35 g available carbohydrate (total carbohydrate minus dietary fiber). The four treatments were quinoa providing 25 g (Q1) or 35 g (Q2) available carbohydrate and white bread providing equivalent carbohydrate amounts (Brd1 and Brd2).

The test portions of dry, uncooked quinoa were weighed to provide 25 g and 35 g of available carbohydrate, corresponding to 36 g and 50 g of quinoa, respectively, based on the nutritional information provided on the product label. Quinoa was prepared by adding the pre-weighed portion of 36–50 g to 250–325 mL of boiling water, respectively, with a pinch of salt. After the water returned to a boil, the heat was reduced to medium, and the quinoa was simmered for 8–9 min with the lid closed to minimize water evaporation. This standardized cooking procedure (water volume, cooking time, and temperature) was used throughout the study to ensure consistent texture and palatability across all test sessions. The quinoa was considered adequately cooked when the grains were tender without being overcooked or undercooked. Immediately after cooking, 10 g of butter (commercially available as individual 10 g portions) was mixed into the quinoa. The cooked quinoa was served within 3–4 min of preparation at a consistent serving temperature and was never reheated or microwaved.

For the control meal, white bread portions, about 2–3 slices, were pre-weighed to provide 25 g or 35 g of available carbohydrate, corresponding to the two test sessions. Individual portions were stored in sealed zip-lock bags at −20 °C until the day of testing. On each test day, the bread was toasted directly from frozen using the same toaster with standardized time and temperature settings. Each serving was provided with 10 g of butter, identical to that used for the quinoa meal. Participants were permitted to season either test meal with a small amount of black pepper, if desired. The participants were required to finish all their test food and water (500 mL of bottled water, Aquafina, PepsiCo Beverages North America, Kuwait City, Kuwait) within 8–10 min.

### 2.3. Study Protocol

A within-subject repeated measures design was used. Participants came for all the sessions, one week apart, at their convenient time between 8.30 and 10.30 am (two hours after waking up) after an overnight fast of 10–12 h. Water was permitted up to 1 h before the scheduled start time. They completed a questionnaire to describe their sleep, stress and activity levels in the previous 24 h. Treatment order was randomized. Female participants were scheduled outside their menstrual phase to minimize hormonal effects on glycemia [42] or feeling of appetite [43].

Capillary blood glucose was measured at baseline and at 15, 30, 45, 60, 90, and 120 min postprandially using a portable glucometer (One Touch Ultra, Life Scan Inc. (Malvern, PA, USA) and Johnson & Johnson Company (New Brunswick, NJ, USA)). Subjective appetite information was collected at the same point as finger prick blood collection using visual analog scales (VASs) as given elsewhere [44]. Subjective appetite is assessed by the feeling of hunger, desire to eat, fullness, and the prospective consumption of the amount of food. A reliable, validated tool used to measure subjective feeling of appetite is the visual analog scale (VAS), which is proven to be reproducible [45,46,47,48]. Briefly, each VAS consists of a 10 cm line anchored at each end with opposing statements for each of the 4 questions: (1) How strong is your desire to eat? (very weak to very strong); (2) How hungry do you feel? (not hungry at all to as hungry as I’ve ever felt); (3) How full do you feel? (not full at all to very full); and (4) How much food do you think you could eat? (nothing at all to a large amount). The subjects marked an “X” on the line to indicate their feelings at that given moment. To quantify subjective appetite sensations, each visual analogue scale (VAS) response is converted into a numerical value by measuring the distance (cm) from the left end of the 10 cm line to the participant’s mark (“X”). Higher scores indicated greater intensity of the sensation being assessed.

Each VAS parameter reflects a distinct dimension of subjective appetite. Hunger represents the physiological drive to eat, desire to eat reflects the motivational or hedonic urge to consume food, prospective food consumption estimates the amount of food an individual believes they could eat, and fullness reflects the sensation of gastric distension and postprandial satiety. Although each VAS parameter can be analyzed independently, an overall average appetite score is calculated by combining the four measures using the established composite appetite score formula.

Average appetite score = [Hunger + Desire to Eat + Prospective Food Consumption + (100 − Fullness)]/4

Each variable contributes equally (25%) to the composite score because they represent complementary aspects of subjective appetite. The fullness score is reversed (100 − fullness) because it is inversely related to appetite, thereby ensuring that all four components are aligned in the same direction before averaging. Consequently, higher composite scores indicate greater appetite, whereas lower scores indicate greater satiety. This composite measure reduces random variability, facilitates comparisons between treatments, and provides a more comprehensive assessment of subjective appetite than any single VAS parameter alone [44,45,46,47,48].

Palatability of the test foods was similarly assessed by a VAS questionnaire for three parameters, texture, aroma and taste, on the same scale as the appetite questionnaire. Physical comfort/gastrointestinal discomfort was assessed by the VAS questionnaire after consumption of the test foods using the question, “How well do you feel?”, with options such as “not well at all” or “very well” given at the opposite ends of the line.

### 2.4. Statistical Analysis

Statistical analyses were performed using SPSS, version 31.00. Two-way repeated-measures ANOVA was used to assess the effects of treatment, time, and their interaction on blood glucose and appetite responses. The effect of treatment on area under the curve (iAUC) for blood glucose and subjective appetite was determined using one-way repeated measures analysis of variance (ANOVA). Incremental area under the curve (iAUC) for blood glucose and net area under the curve (nAUC) for change in appetite were calculated using the trapezoid rule for 0 to 120 min. Post hoc comparisons were conducted using Tukey’s test. Pearson correlation coefficients were used to assess associations between glycemic and appetite responses. Statistical significance was set at *p* ≤ 0.05. Figures were made using GraghPad Prism, version 11.00.

## 3. Results

### 3.1. Study Participants

**Scheme 1:**
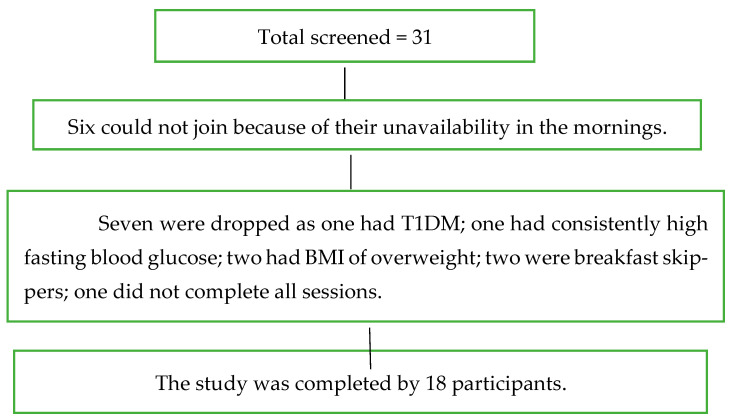
Subjects’ elimination and completion of the study.

After screening by the inclusion/exclusion criteria for eligibility to participate in this study, only 18 out of 31 students could complete the study. Detailed information on the participants is given in the flow diagram below (Scheme 1).

### 3.2. Postprandial Blood Glucose Response

Postprandial blood glucose concentrations were significantly affected by treatment (*p* < 0.001), time (*p* < 0.01), and treatment × time interaction (*p* = 0.021). All test foods produced a rise in blood glucose from baseline; however, the magnitude and temporal pattern of responses differed markedly between quinoa and white bread.

**Figure 1:**
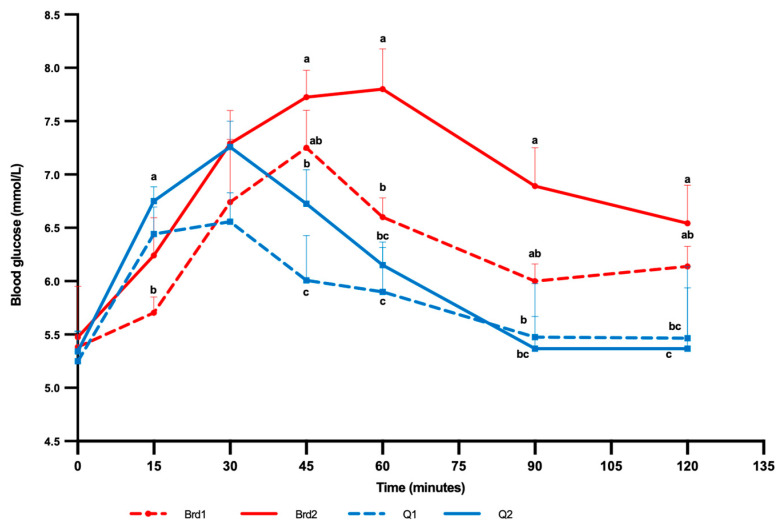
Effect of quinoa and bread treatment on blood glucose response over 120 min. The data is presented as mean ± SD. Different superscript letters show significant differences among the treatments at the same time point. Statistical significance was set at *p* ≤ 0.05.

Despite equivalent available carbohydrate content, quinoa elicited lower and less sustained glycemic responses than white bread. Postprandial glucose concentrations peaked earlier and declined faster following quinoa consumption than after white bread. Following the peak, glucose concentrations dropped to the baseline level after both quinoa treatments, whereas elevated glucose levels persisted throughout the 120 min period after bread consumption, particularly following Brd2 (Figure 1).

The data is presented as mean ± SD. Different superscript letters show significant differences among the treatments at the same time point. Statistical significance was set at *p* ≤ 0.05.

**Figure 2:**
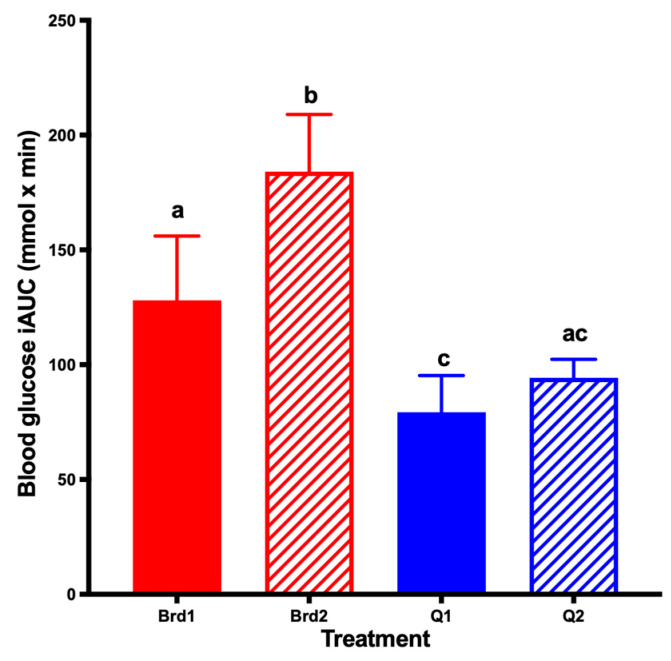
Effect of quinoa and bread treatment on blood glucose iAUC over 120 min. The data is presented as mean ± SD. Different superscript letters show significant differences among the treatments. Statistical significance was set at p ≤ 0.05.

Incremental area under the curve (iAUC) analysis confirmed these observations. Blood glucose iAUC over 120 min was significantly lower after Q1 and Q2 compared with Brd1 and Brd2 (F = 4.736; *p* = 0.006; η^2^ = 0.244; 95% CI = 0.26, 0.400), with no significant difference between the two quinoa doses (Figure 2). Increasing the carbohydrate load from 25 g to 35 g resulted in a marked increase in glycemic exposure for bread but not for quinoa.

The data is presented as mean ± SD. Different superscript letters show significant differences among the treatments. Statistical significance was set at *p* ≤ 0.05.

### 3.3. Subjective Appetite Responses

Subjective appetite scores decreased immediately following consumption of all test foods, reaching a nadir at 15 min, followed by a gradual increase over time. However, appetite recovery differed significantly among treatments.

Subjective appetite responses over 120 min following consumption of the test foods were evaluated through visual analog scales (VASs) using the four assessment questions including desire to eat, hunger, prospective food consumption and fullness (Table 1).

All treatments showed a reduction in desire to eat immediately after consumption, followed by a gradual increase over time. However, significant differences among treatments emerged at 60, 90, and 120 min (*p* = 0.05, 0.04, and 0.05, respectively). The Q2 treatment consistently produced the lowest desire-to-eat scores at these later time points. At 120 min, participants consuming Q2 reported markedly lower desire to eat than those consuming Brd1.

Hunger ratings decreased after meal consumption in all treatments and progressively increased thereafter. Although no statistically significant differences were observed at most time points, a borderline significant difference was observed at 120 min (*p* = 0.06). Q2 tended to maintain lower hunger ratings throughout the postprandial period compared with the bread treatments.

Participants’ perception of how much food they could eat decreased after consumption of all treatments and gradually increased over time. Significant differences were observed at 120 min (*p* = 0.05). The Q2 treatment resulted in the lowest ratings at the final time point, indicating reduced perceived capacity for further food intake and suggesting greater satiety compared with Brd1 and Brd2.

Fullness scores increased markedly after meal consumption in all treatments and gradually declined over the 120 min period. Significant differences were observed at 120 min (*p* = 0.03). Q2 produced the highest fullness ratings at the end of the study period, while Brd1 showed the lowest fullness response.

From the scores of the four subjective appetite questions, average appetite responses over 120 min were calculated following consumption of the bread and quinoa treatments. All treatments initially reduced appetite sensations after intake; however, the magnitude and duration of these effects differed among treatments.

**Figure 3:**
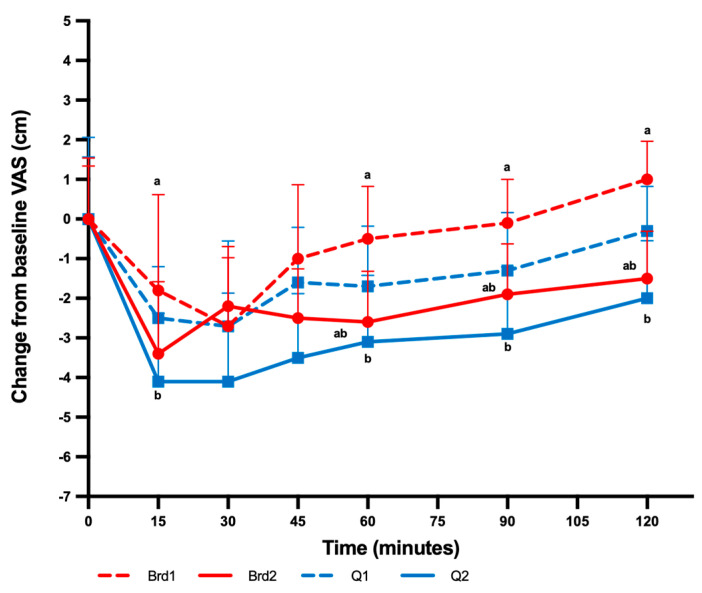
Effect of quinoa and bread treatment on subjective appetite response over 120 min. The data is presented as mean ± SD. Different superscript letters show significant differences among the treatments at the same time point. Statistical significance was set at *p* ≤ 0.05.

The Q2 treatment demonstrated a stronger appetite-suppressing effect, characterized by lower desire-to-eat and hunger ratings and higher fullness ratings during the later postprandial period. Significant differences among treatments were particularly evident at 120 min, where Q2 maintained greater satiety compared with Brd1. In contrast, Brd1 showed a more rapid return of appetite sensations, indicating weaker satiety effects (*p* < 0.05) (Figure 3).

**Figure 4:**
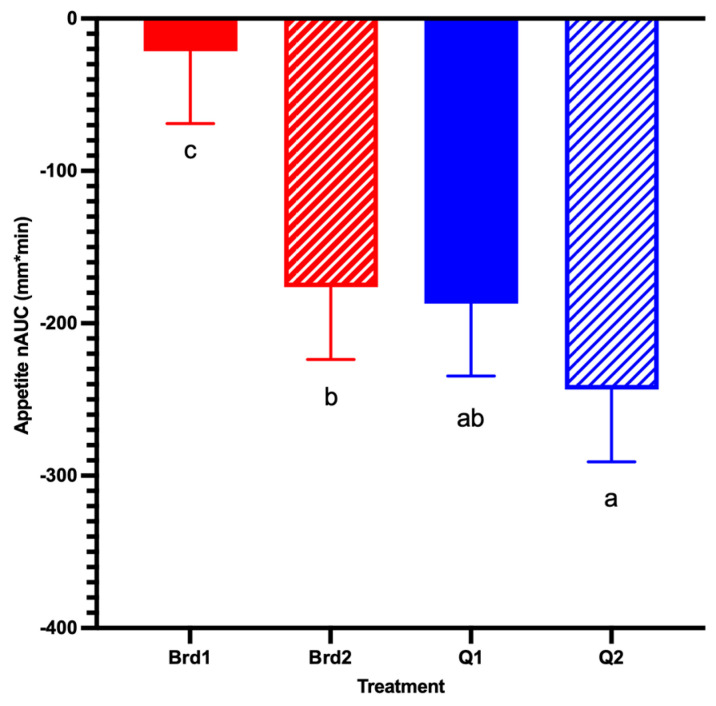
Effect of quinoa and bread treatment on subjective appetite nAUC over 120 min. The data is presented as mean ± SD. Different superscript letters show significant differences among the treatments. Statistical significance was set at *p* ≤ 0.05.

The net incremental area under the curve (nAUC) for subjective appetite responses over 120 min (Figure 4) shows that both Q1 and Q2 produced the most negative nAUC value, followed by Brd2, while Brd1 showed the least negative response. More negative nAUC values indicate a greater overall suppression of appetite (*p* < 0.05).

### 3.4. Relationship Between Glycemic and Appetite Responses

Blood glucose iAUC was positively correlated with appetite nAUC (r = 0.57; *p* < 0.001). This relationship remained significant after controlling for treatment effects, suggesting a physiological link between glycemic excursions and appetite regulation.

### 3.5. Palatability and Gastrointestinal Tolerance

There were no significant differences among treatments in sensory attributes, including aroma, taste, and texture. Gastrointestinal discomfort scores were low and did not differ between quinoa and bread at either dose level (Table 2), indicating good tolerability and acceptability of quinoa consumption.

## 4. Discussion

The present study demonstrates that acute consumption of cooked quinoa elicits significantly lower postprandial glycemic excursion and improved subjective appetite control compared with white bread when matched for available carbohydrate in healthy young female volunteers. These findings support the hypothesis that quinoa represents a favorable carbohydrate source with potential benefits for glycemic regulation and satiety.

### 4.1. Glycemic Effect of Quinoa

Despite providing equivalent amounts of available carbohydrate, quinoa and white bread produced distinct postprandial glycemic responses. Quinoa consumption resulted in earlier and lower glycemic peaks, followed by a more rapid return toward baseline, whereas white bread produced a more sustained elevation in blood glucose throughout the 120 min postprandial period. Consequently, both quinoa treatments produced significantly lower incremental area under the curve (iAUC) values than white bread, with no significant difference observed between the two quinoa doses. These findings suggest that the lower dose of 36 g cooked quinoa (providing 25 g available carbohydrate) was as effective as the higher dose of 50 g (providing 35 g available carbohydrate) in attenuating postprandial glycemic excursions in healthy young women.

The present findings differ from most previous studies investigating quinoa supplemented as dietary intervention, with the exception of one study reporting improved glycemic responses following consumption of bread supplemented with 20 g quinoa [25]. In contrast, supplementation with 15 g quinoa in biscuits consumed daily for four weeks [29], approximately 20 g quinoa incorporated into cereal bars [27], or a similar amount added to bread [26] did not significantly alter glycemic responses in healthy adults. However, studies employing larger quinoa doses, longer intervention periods, or combining quinoa consumption with lifestyle modifications have reported beneficial metabolic effects. For example, daily consumption of 25 g cooked quinoa combined with regular exercise for eight weeks significantly reduced fasting blood glucose concentrations and appetite ratings in adults with type 2 diabetes [49]. Likewise, consumption of approximately 50 g quinoa in conjunction with caloric restriction improved insulin sensitivity in overweight adults [31], whereas long-term consumption of 100 g/day quinoa or a total replacement for rice and wheat-based staple foods improved glycemic control and insulin resistance in individuals with impaired glucose tolerance [32,33,34,35].

Collectively, these findings suggest that the metabolic benefits of quinoa are influenced not only by the amount consumed but also by intervention duration, participants’ metabolic status, and concurrent lifestyle modifications. In the present acute dose–response study, both quinoa treatments, providing 25 or 35 g of available carbohydrate, elicited significantly lower and less sustained postprandial glycemic responses than white bread, suggesting slower glucose absorption and more efficient postprandial glucose handling. Notably, the absence of significant differences in glucose iAUC between the two quinoa doses indicates that the lower dose may be sufficient to confer comparable acute glycemic benefits in healthy young women.

The favorable glycemic response observed following quinoa consumption is likely attributable to its unique starch structure and nutrient composition. Compared with high-glycemic staple cereals such as rice and wheat [50], quinoa starch contains lower proportions of rapidly digestible starch and higher levels of non-starch polysaccharides [16]. In addition, quinoa starch is composed of very small granules embedded within a compact grain matrix, making it less susceptible to enzymatic hydrolysis. These structural characteristics may delay carbohydrate digestion and slow glucose release into the circulation, thereby attenuating postprandial glycemic excursions. The lower postprandial glucose response observed in the present study is unlikely to be explained by resistant starch formation, as the freshly cooked quinoa was consumed immediately after preparation. Resistant starch is primarily formed during cooling through starch retrogradation. Quinoa amylopectin contains a high proportion of short branch chains and relatively few long branch chains, resulting in limited gelatinization and slower retrogradation compared with many cereal starches, thereby reducing the formation of resistant starch after cooking [14,51,52,53].

In addition to its carbohydrate profile, quinoa contains numerous bioactive compounds that may contribute to glycemic regulation. Polyphenols, flavonoids, saponins, and antioxidant vitamins present in quinoa have been associated with improved glucose metabolism and reduced carbohydrate digestion. Zhou et al. [54] reported that peptides generated during quinoa protein hydrolysis may inhibit α-amylase activity through intermolecular interactions, thereby reducing starch digestion. Similarly, phenolic compounds isolated from quinoa have demonstrated inhibitory effects against both α-amylase and α-glucosidase, potentially delaying intestinal glucose absorption and contributing to the lower glycemic response observed in the present study [55,56,57]. Experimental studies have also suggested that certain bioactive phytochemicals in quinoa, including alkaloids and polyphenols, possess insulinotropic properties that may stimulate insulin secretion and enhance peripheral glucose uptake [56,57,58]. Furthermore, activation of AMP-activated protein kinase (AMPK) signaling pathways by these phytochemicals has been reported to promote glucose utilization in peripheral tissues, while flavonoids may suppress hepatic gluconeogenesis, thereby contributing to improved glucose homeostasis [57,59]. Collectively, these findings suggest that the favorable glycemic effects of quinoa are likely mediated through the synergistic actions of slowly digestible starch, dietary fiber, intact cellular structure, protein, and bioactive phytochemicals, which together may delay carbohydrate digestion and glucose absorption while enhancing glucose utilization and overall glycemic regulation.

### 4.2. Satiety Effect of Quinoa

In parallel with its favorable effects on postprandial glycemia, quinoa consumption produced greater suppression of subjective appetite than white bread at both carbohydrate-matched doses (36 and 50 g cooked quinoa, providing 25 and 35 g available carbohydrate, respectively). Following quinoa consumption, participants reported a lower desire to eat, reduced prospective food intake, lower hunger ratings, and significantly greater feelings of fullness. The more negative net area under the curve (nAUC) values observed after quinoa consumption indicate greater appetite suppression and prolonged satiety compared with white bread. Furthermore, the positive correlation between blood glucose iAUC and appetite nAUC suggests that attenuation of postprandial glycemia may contribute to improved appetite regulation. These findings are consistent with a previous human study that also reported enhanced satiety following quinoa consumption [49]. Although human studies investigating the effects of quinoa on appetite regulation remain limited, evidence from animal studies suggests that quinoa may modulate appetite-related hormones, including ghrelin, leptin, and cholecystokinin, which play important roles in the regulation of hunger and satiety [20,22,23,60].

The relatively high dietary fiber and protein contents of quinoa are likely to contribute to both its glycemic and satiety-promoting effects. Dietary fiber has consistently been associated with delayed gastric emptying, slower glucose absorption, improved insulin sensitivity, and enhanced satiety [32,48,59,61,62,63]. In particular, the insoluble fiber components of quinoa, including polysaccharides containing galactose, galacturonic acid, and xylose, may increase gastrointestinal viscosity and prolong gastric transit time, thereby slowing nutrient absorption, enhancing satiety, and attenuating postprandial glycemic responses [16,49]. In addition, quinoa contains approximately 14–16% high-quality protein with a well-balanced amino acid profile, which may further enhance satiety through delayed gastric emptying and modulation of appetite-regulating hormones [64].

Importantly, quinoa was well accepted and tolerated at both dose levels, with no significant differences in palatability or gastrointestinal discomfort compared with white bread. This finding supports the practical application of quinoa as a functional carbohydrate alternative, as sensory acceptability and gastrointestinal tolerance are key determinants of long-term dietary adherence. Beyond its favorable nutritional composition, quinoa is naturally gluten-free and possesses remarkable agronomic resilience to drought, salinity, and adverse environmental conditions. These characteristics have led to its recognition as an important crop for sustainable food systems and global food security. Recent reviews have further highlighted quinoa as a promising functional food capable of addressing both nutritional deficiencies and the growing burden of chronic non-communicable diseases [10,13,53].

Overall, the findings of this acute study demonstrate that consumption of quinoa providing 25–35 g of available carbohydrate elicits more favorable postprandial glycemic and satiety responses than an equivalent amount of carbohydrate from white bread. These results support the potential of quinoa as a practical functional carbohydrate alternative that may contribute to improved metabolic health and appetite regulation. Notably, these benefits were achieved with relatively modest amounts of quinoa, suggesting that its incorporation into the daily diet may provide metabolic advantages without requiring complete replacement of commonly consumed staple foods such as rice or wheat.

## 5. Strengths and Limitations

The randomized crossover design is a major strength of this preliminary acute study, as each participant served as her own control, thereby minimizing inter-individual variability and reducing the influence of potential confounding factors. Another important strength was the use of test meals matched for available carbohydrate content, allowing direct comparison of the physiological effects of quinoa and white bread independently of carbohydrate quantity. The standardized preparation of quinoa and white bread, controlled testing conditions, and supervised meal consumption further enhanced the internal validity of the study. Subjective appetite was assessed using validated visual analog scales (VASs), facilitating comparison with previous studies and ensuring methodological reproducibility. In addition, the study population was relatively homogeneous with respect to age, sex, body mass index, health status, medication use, and breakfast consumption habits, thereby reducing biological variability. The sample size was estimated based on previous intervention studies and was adequately powered to detect differences among the treatments. Finally, administering quinoa and white bread as individual test foods, rather than as components of mixed meals, minimized the influence of other dietary constituents such as fat, protein, fiber, and phytochemicals on postprandial glycemic and appetite responses.

Several limitations should also be acknowledged. First, the study included only healthy young female adults, which may limit the generalizability of the findings to males, older adults, or individuals with obesity, prediabetes, or type 2 diabetes. Second, appetite was assessed using subjective VAS ratings alone. Although VAS is a well-validated and widely accepted tool for measuring appetite sensations, these ratings reflect participants’ perceptions at a given time and may be influenced by physiological, psychological, and environmental factors. Future studies should therefore combine subjective appetite assessment with objective measures, including subsequent energy intake and circulating appetite-regulating hormones such as ghrelin, leptin, glucagon-like peptide-1 (GLP-1), cholecystokinin (CCK), and peptide YY (PYY), to provide a more comprehensive evaluation of satiety.

As this was an acute feeding study, the findings cannot be extrapolated to the long-term metabolic effects of regular quinoa consumption. Well-designed randomized controlled trials of longer duration are required to determine whether the observed improvements in postprandial glycemia and satiety translate into sustained benefits for body weight regulation and cardiometabolic health in populations at increased risk of obesity and type 2 diabetes. Furthermore, although quinoa is generally regarded as safe, the long-term safety of consuming larger quantities should be further evaluated. Naturally occurring phytochemicals, including saponins and alkaloids, warrant continued investigation to establish their long-term safety and potential effects on nutrient bioavailability when quinoa is consumed regularly at high intakes.

## 6. Conclusions

This preliminary randomized crossover study demonstrates that cooked quinoa elicits significantly lower postprandial glycemic responses and greater subjective satiety than white bread when matched for available carbohydrate in healthy young female adults. Both quinoa doses (25 g and 35 g available carbohydrate) significantly reduced postprandial glycemic exposure and enhanced appetite suppression without compromising palatability or gastrointestinal tolerance. The comparable glycemic responses observed following the two quinoa doses suggest that the lower dose (36 g dry quinoa, providing 25 g available carbohydrate) may be sufficient to confer acute metabolic benefits.

These findings support the potential of cooked quinoa as a functional carbohydrate alternative that may improve postprandial glycemic control and appetite regulation. Incorporating moderate amounts of quinoa into habitual diets may represent a practical dietary strategy for populations with a high intake of refined carbohydrates and an increasing burden of obesity and type 2 diabetes. Future randomized controlled trials incorporating measurements of insulin, incretin and appetite-regulating hormones, together with longer intervention periods in metabolically at-risk populations, are needed to confirm these findings and further elucidate the underlying mechanisms.

## Figures and Tables

**Table 1:** Participants’ responses (mean ± SD) for the four subjective appetite questions measured on a visual analog scale (10 cm long).

Q1: How Strong Is Your Desire to Eat?							
Time (min)	0	15	30	45	60	90	120
Brd1	7.5 ± 1.5	3.7 ± 2.7	4.8 ± 2.2	6.6 ± 1.8 ^a^	7.0 ± 1.7 ^a^	7.4 ± 1.7 ^a^	8.5 ± 0.9 ^a^
Brd2	7.9 ± 1.8	5.3 ± 1.2	5.9 ± 1.3	5.7 ± 1.3 ^ab^	5.6 ± 1.7 ^b^	6.3 ± 1.9 ^ab^	6.7 ± 1.9 ^ab^
Q1	7.5 ± 1.7	5.3 ± 2.4	4.8 ± 2.2	5.9 ± 1.8 ^ab^	5.8 ± 1.0 ^b^	6.2 ± 1.5 ^ab^	7.2 ± 1.5 ^ab^
Q2	7.7 ± 1.9	3.6 ± 2.3	3.6 ± 2.1	4.2 ± 1.8 ^b^	4.2 ± 1.9 ^c^	4.3 ± 2.0 ^b^	5.1 ± 1.5 ^b^
*p*-value	0.79	0.33	0.75	0.08	0.05	0.04	0.05
Q2: How Hungry Do You Feel?							
Brd1	7.3 ± 2.0	4.1 ± 2.8	5.1 ± 2.3	5.7 ± 1.9	6.1 ± 2.1	6.5 ± 1.5	7.2 ± 1.5
Brd2	7.4 ± 1.4	4.9 ± 2.3	4.8 ± 2.4	5.9 ± 1.4	5.6 ± 2.1	6.6 ± 1.3	7.7 ± 1.4
Q1	7.5 ± 2.0	5.3 ± 1.4	5.1 ± 2.3	6.2 ± 1.5	6.0 ± 1.9	6.8 ± 2.6	7.4 ± 2.0
Q2	7.3 ± 3.1	5.4 ± 2.9	5.5 ± 2.4	5.5 ± 1.8	5.8 ± 2.0	6.1 ± 2.0	6.2 ± 1.6
*p*-value	0.95	0.41	0.56	0.56	0.37	0.81	0.06
Q3: How Much Food Do You Think You Could Eat?							
Brd1	6.5 ± 1.9	4.1 ± 2.2	4.7 ± 2.2	5.5 ± 2.0	6.4 ± 1.1	6.5 ± 1.5	7.3 ± 1.7 ^a^
Brd2	6.8 ± 2.0	3.9 ± 2.2	4.8 ± 1.2	5.1 ± 1.2	5.5 ± 1.1	6.5 ± 1.4	7.1 ± 1.6 ^a^
Q1	6.5 ± 2.1	4.8 ± 1.9	4.5 ± 2.1	5.4 ± 2.0	5.5 ± 1.8	5.9 ± 1.4	6.1 ± 1.1 ^ab^
Q2	7.3 ± 2.5	5.4 ± 2.5	5.6 ± 1.7	5.6 ± 1.5	5.7 ± 1.7	5.6 ± 1.5	5.5 ± 1.1 ^b^
*p*-value	0.55	0.51	0.32	0.62	0.28	0.21	0.05
Q4: How Full Do You Feel?							
Brd1	2.2 ± 1.6	5.0 ± 2.9	3.8 ± 2.9	4.4 ± 2.1	4.5 ± 1.7	3.8 ± 2.0	2.3 ± 0.9 ^a^
Brd2	1.8 ± 1.3	5.8 ± 2.5	5.8 ± 2.5	5.1 ± 1.8	4.8 ± 1.7	4.5 ± 1.8	4.2 ± 1.9 ^b^
Q1	2.0 ± 1.6	5.4 ± 1.5	4.9 ± 2.4	4.7 ± 2.4	4.3 ± 2.7	4.3 ± 2.2	4.3 ± 1.2 ^b^
Q2	1.8 ± 1.4	7.1 ± 1.6	6.9 ± 2.7	5.4 ± 2.4	5.2 ± 1.8	5.0 ± 1.8	5.0 ± 1.8 ^bc^
*p*-value	0.68	0.18	0.18	0.51	0.82	0.51	0.03

The data is presented as mean ± SD. Different superscript letters show significant differences among the treatments within the columns. Statistical significance was set at *p* ≤ 0.05.

**Table 2:** Participants’ sensory and gastrointestinal responses to the test foods (mean ± SD).

Variables	Aroma	Taste	Texture	Stomach Discomfort
Bread 1	7.47 ± 0.4	8.11 ± 0.7	7.92 ± 2.2	1.13 ± 2.1
Bread 2	7.37 ± 0.7	8.12 ± 1.2	7.11 ± 1.5	2.21 ± 1.9
Quinoa 1	7.62 ± 0.8	8.22 ± 0.9	7.14 ± 1.6	2.11 ± 1.4
Quinoa 2	8.19 ± 0.9	7.99 ± 1.2	7.45 ± 1.3	2.21 ± 1.3

## Data Availability

The original contributions presented in the study are included in the article, further inquiries can be directed to the corresponding author.
